# A Comprehensive Approach to Evaluate Durum Wheat–Faba Bean Mixed Crop Performance

**DOI:** 10.3389/fpls.2022.733116

**Published:** 2022-03-23

**Authors:** Stefano Tavoletti, Ariele Merletti

**Affiliations:** Dipartimento di Scienze Agrarie, Alimentari e Ambientali, Università Politecnica delle Marche, Ancona, Italy

**Keywords:** durum wheat, *Vicia faba* minor, breeding for intercropping, principal component analysis, land equivalent ratio

## Abstract

Plant breeding for intercropping is lagging because most varieties currently available in the market are selected for sole cropping systems. The present study analyzed the response of durum wheat (12 varieties) and faba bean (3 varieties) in pure and mixed cropping. Field trials were conducted in 2019 and 2020. The performance of each variety in mixed and pure cropping was evaluated using both univariate and multivariate analyses of the grain yield and land equivalent ratio (LER). For durum wheat, grain protein content was also evaluated. Durum wheat varieties were characterized by good performance in both years, whereas faba bean varieties were more affected by the growing season, suggesting that much breeding effort is warranted to improve the latter as a pure and mixed crop. Moreover, the relative performance of all varieties was affected by their combination in mixed cropping, as evaluated based on the ratio (LERratio) between LER for wheat (LERw) and LER for faba bean (LERfb). To further evaluate the overall performance of wheat and faba bean in mixed cropping, total yield, LERtotal (LERw + LERfb), and ln(LERratio) were subjected to principal component and cluster analyses. The first principal component combined the total yield and LERtotal in a single index of the overall performance of each mixed crop combination. The second principal component, based on ln(LERratio), highlighted the relative performance of varieties in each mixed crop combination. The proposed multivariate approach can be applied in the breeding programs for intercropping to identify variety combinations based on crop performance and the relative importance of the proportion of cereal and legume grains in the total harvest.

## Introduction

Intercropping is the cultivation of different crops in the same field at the same time and it has been recognized as an alternative to pure crops for the development of more sustainable agricultural systems (Malézieux et al., 2009; Lithourgidis et al., 2011; Costanzo and Bàrberi, 2014; Brooker et al., 2015; Martin-Guay et al., 2018; Li et al., 2020; Maitra et al., 2021). Much attention has been paid to cereal–grain legume intercropping, including both cool- and warm-season crops (Hauggaard-Nielsen et al., 2008; Bedoussac et al., 2015). Among the cool-season crops, research has mainly focused on bread wheat (*Triticum aestivum* L.), durum wheat (*Triticum turgidum* ssp. *durum* Desf. Husn.), and barley (*Hordeum vulgare* L.) as cereals intercropped with faba bean (*Vicia faba* L.) and pea (*Pisum sativum* L.) as grain legumes (Bedoussac and Justes, 2010a,b; Sahota and Malhi, 2012; Abdel-Wahab and El Manzlawy, 2016; Galanopoulou et al., 2019; Kammoun et al., 2021; Nelson et al., 2021). In pure cropping, these crops are grown in dense stands (Hauggaard-Nielsen and Jensen, 2001), with a higher plant density for cereals (300–400 plants m^–2^) than for grain legumes (on average, 35–45 plants m^–2^ for faba bean and 80–90 plants m^–2^ for pea). Typically, in intercropping, the cereal–legume combinations are grown as mixed crops, with the plants of the two crops planted as a mixture in the field without a specific row arrangement (Aziz et al., 2015; Layek et al., 2018). Under such growing conditions, strong interspecific interactions occur at both the aerial and root levels (Bedoussac and Justes, 2011; Pivato et al., 2021). Thus, morphophysiological traits characterizing each component warrant attention because of their importance to the overall performance of the mixed crops.

Increased biodiversity due to intercropping is advantageous for soil health (Wahbi et al., 2016) as well as nitrogen (N) and P bioavailability (Fustec et al., 2010; Li et al., 2016; Kaci et al., 2018; Ingraffia et al., 2019). In particular, the complementary use of N resources, that is, mineral N for cereals and atmospheric N_2_ for legumes, entails lower N fertilization, which reduces carbon dioxide (CO_2)_ emissions and N losses, ultimately lowering the inputs in more sustainable agricultural systems (Ghaley et al., 2005; Pelzer et al., 2012; Jensen et al., 2020). Simultaneously, compared with legume pure crops, cereal–grain legume mixed cropping enhances the competitive ability of crops against weeds through the allelopathic effects of cereals, reducing the use of herbicides, offering further opportunities to increase grain legume production through low-input conventional and organic farming systems (Wu et al., 2001; Agegnehu et al., 2008; Corre-Hellou et al., 2011; Zander et al., 2016; Bybee-Finley and Ryan, 2018). Moreover, mixed cropping can reduce the damage caused by diseases and parasitic plants (*Orobanche* spp.), which represent the major hurdles to increase grain legume cultivation, particularly in the Mediterranean countries (Fernández-Aparicio et al., 2011, 2016; Karkanis et al., 2018). Overall, intercropping is a reliable alternative to intensive agricultural systems, which rely upon pure crops, for lowering the environmental impacts of agriculture through reduced use of agrochemicals, increased biodiversity within cultivated fields, and enhanced ecosystem services while increasing the crop yield and ensuring stable production (Malézieux et al., 2009; Bedoussac et al., 2015; Raseduzzaman and Jensen, 2017; Hawes et al., 2019; Weih et al., 2021).

In the process of transition toward more sustainable agricultural systems, plant breeding may play a vital role in facilitating the transition from pure cropping to intercropping (Lulie, 2017; Raseduzzaman and Jensen, 2017; Fung et al., 2019). However, selection for sole cropping cannot produce the best genotypes for intercropping, and alternative breeding schemes must be established for intercropping (Lithourgidis et al., 2011; Gaba et al., 2015; Litrico and Violle, 2015; Haug et al., 2021). Recurrent selection (Sampoux et al., 2020), incomplete factorial designs (Haug et al., 2021), and genomic selection (Bančič et al., 2021) have recently been proposed as the strategies for mixed crop breeding. Moreover, models to identify traits that are the most closely linked to mixed cropping performance have been developed (Berghuijs et al., 2020; Louarn et al., 2020).

In the present study, we explored the mixed cropping of durum wheat and faba beans (*Vicia faba* L. var minor Beck). Durum wheat is mainly cultivated for human consumption, whereas small-seed faba beans are primarily used as a protein concentrate in feedstock (Mariotti et al., 2018). While there has been marked progress in terms of plant breeding for durum wheat (Royo et al., 2009; Beres et al., 2020; Xynias et al., 2020), genetic selection for faba bean, although applied effectively (Maalouf et al., 2019; Carrillo-Perdomo et al., 2020), has been limited. These discrepancies are reflected in the performance of cereal and grain legumes, particularly yield stability; as such, faba bean yield is much more variable across years than durum wheat yield (Annicchiarico et al., 2019).

In two successive years, mixed crop combinations of durum wheat and faba bean varieties, which are commonly cultivated in central Italy, were evaluated to (1) compare the performance of mixed crop combinations with that of sole crops, (2) compare the effects of mixed and sole cropping on durum wheat grain protein content, and (3) develop an approach based on multivariate analyses (principal component and cluster analyses) for the characterization of the overall performance of durum wheat–faba bean mixed crop combinations. Although based on commercial varieties, the results of the present study provide information that could be applied to gather a comprehensive evaluation of durum wheat-faba bean combinations in breeding programs for intercropping.

## Materials and Methods

### Field Trials

Two field trials were set at the experimental station of the Università Politecnica delle Marche (Italy) on December 10, 2018 (trial 1: 43°31′54.41″N and 13°22′00.93″E) and January 22, 2020 (trial 2: 43°32′41.09″N, 13°21′34.13″E). Regarding crop rotation, the preceding crops were sunflower and barley for trials 1 and 2, respectively. The high level of precipitation (Supplementary Figure 1) registered from September to December 2019 delayed the sowing time for trial 2. However, delayed sowing is rather common in this area of central Italy and the two sowing times in the present field trials represent a normal trend in the local agricultural systems.

Both field trials were conducted in silty–clayey soils, with the relative sand, silt, and clay content of 11.7, 42.4, and 45.9% in trials 1 and 17.4, 43.3, and 39.3% in trial 2, respectively. Soils in trials 1 and 2 were characterized by similarly high pH (8.13 and 8.14), very high (130 g kg^–1^), and high (55 g kg^–1^) active calcium carbonate content, moderate (11.9 g kg^–1^) and low (9.5 g kg^–1^) available P, high available K (305 and 295 g kg^–1^), low (16.9 g kg^–1^), and moderate (20.6 g kg^–1^) organic matter content, and moderate total N content (1.20 and 1.15 g kg^–1^), respectively.

Mixed crops were sown with intermixed cereal and faba bean seeds in a single step. Therefore, a sowing depth of 3 cm was used, which was a compromise between the sowing depths of 2–3 cm for wheat and 3–5 cm for small-seeded faba beans suggested for our pedo-climatic conditions.

Different levels of N fertilization were applied to durum wheat pure (180 kg N⋅ha^–1^) and mixed crops (90 kg N⋅ha^–1^) because the amount of N fertilizer (urea, 46%) was set based on durum wheat seed density. Pure faba bean crops were not fertilized, according to the local farming practices.

For both trials, a randomized complete block design with four replicates was applied and each plot comprised eight rows (length, 5 m) spaced 15 cm apart.

### Durum Wheat and Faba Bean Varieties

A total of 12 durum wheat and three faba bean varieties, cultivated as pure crops in central Italy and representing a wide range of varieties in terms of grain yield and quality, were included in the field trials to assess their responses to mixed cropping (Supplementary Table 1). A total of 11 durum wheat varieties were chosen because of their relatively good performance across years, whereas Aureo was included because of its very high protein content, despite its lower yield than that of the other varieties available on the market. Three faba bean varieties (Chiaro di Torrelama, Prothabat69, and Rumbo) were included in 2019 (Supplementary Table 1), whereas Rumbo was not included in 2020 because the seeds of this variety were not available in that growing season. Therefore, results involving Rumbo in 2019 are summarized in Supplementary Material.

Since a preliminary trial performed in 2017 suggested that the 50:50 replacement ratio was suboptimal, mixed crops were sown at a seed density ratio of 50 and 65% for durum wheat and faba bean, respectively (additive design, seed density expressed as a percentage of the respective pure crops). A factorial design was applied to evaluate 36 and 24 mixed crop combinations in 2019 and 2020, respectively. In July 2019 and 2020, each plot was harvested using a Wintersteiger Delta combine harvester for field experimental trials.

### Traits Evaluated

The total grain yield (Mg ha^–1^) of pure and mixed crops was measured. Wheat and faba bean grains from mixed cropping were separated *via* sieving to determine the yield (Mg ha^–1^) of each crop. Moreover, durum wheat grain protein content (%) was evaluated.

For each mixed crop combination, the land equivalent ratio (LER) of each crop and total LER were calculated as follows (Vandermeer, 1989; Bedoussac et al., 2015):

LER for wheat (LER_w_) = Durum wheat yield as a mixed crop/durum wheat yield as a pure crop

LER for faba bean (LER_fb_) = Faba bean yield as a mixed crop/faba bean yield as a pure crop

Total LER (LER_total_) = LER_w_ + LER_fb_

Intercropping is considered to present better land-use efficiency than sole cropping when LER_total_ exceeds one. Since the partial LER value (LER_w_ and LER_fb_) represents the relative performance of a specific variety in terms of its performance as a pure crop, the LER_ratio_ = LER_w_/LER_fb_ was used as an index of the relationship between the relative performances of the two crops in each mixed crop combination. The LER_ratio_ recalls the competitive ratio (CR) proposed by Willey and Rao (1980), but the ratio LERw/LERfb was not corrected for the proportions in which the crops were initially sown, as applied for the CR coefficient.

The rationale behind the use of LER_ratio_ was as follows: LERratio = 1 indicates that LER_w_ = LERfb, therefore both crops show equal relative performance in mixed cropping. In contrast, LERratio > 1, that is LERw > LERfb, indicates that durum wheat performed better than faba bean in mixed cropping, or *vice versa* if LERratio < 1, that is LERw < LERfb.

For data analysis, the raw data of LER_ratio_ were log-transformed (natural log transformation, ln) to obtain ln(LER_ratio_). The ln-transformation was needed to overcome the heteroscedasticity of residual errors due to the different ranges of variation of raw LER_ratio_ data when LER_ratio_ > 1 or when LER_ratio_ < 1 (0 < LER_ratio_ < 1). Moreover, after ln-transformation, complementary situations of cereal and legume performance in mixed cropping share the same absolute ln(LERratio) but with the opposite sign. For instance, if the performance of durum wheat is two times higher than that of faba bean, LER_ratio_ = 2/1 = 2; if the performance of faba bean is two times higher than that of durum wheat, LER_ratio_ = 1/2 = 0.5. Following ln-transformation, the ln(LER_ratio_) values are ln(2) = + 0.693 and ln(0.5) = −0.693. After ANOVA and mean comparisons based on ln-transformed data, the ln(LER_ratio_) means were subsequently transformed to LER_ratio_ ratios using the exponential (exp) function.

### Univariate Data Analyses

Analysis of variance (ANOVA) was performed on the data of only Chiaro di Torrelama and Prothabat69 as faba bean varieties since they were included in both years. Different ANOVA fixed models were applied based on the results of the Shapiro–Wilk and Bartlett’s tests, applied to assess the normal distribution of residuals and homogeneity of variances, respectively.

Pure crop yield (Mg ha^–1^), including both durum wheat and faba bean varieties, was analyzed using the following ANOVA model:


(Model1) $${\text{y}}_{\text{ijk}}=\text{μ}+{\text{α}}_{\text{i}}+{\text{ρ}}_{\text{j}\left(\text{i}\right)}+{\text{β}}_{\text{k}}+{\text{αβ}}_{\text{ik}}+{\text{ε}}_{\text{ijk}}$$

where y_ijk_ = pure crop yield; μ = overall mean; α_i_ = year *effect* (i = 1,2);ρ_j(i)_ = blocks (j = 1,…,4) nested within the year; β_k_ = pure crop effect (k = 1,…,14; 12 wheat + 2 faba bean varieties); αβ_ik_ = year × pure crop interaction; and ε_ijk_ = residual error.

For mixed crops, the durum wheat yield (Mg ha^–1^), total yield (Mg ha^–1^), and ln(LER_ratio_) were analyzed using the following ANOVA model, including year as the main effect and its first- and second-order interactions:


(Model2) $${\text{y}}_{\text{ijkl}}=\text{μ}+{\text{α}}_{\text{i}}+{\text{ρ}}_{\text{j}\left(\text{i}\right)}+{\text{β}}_{\text{k}}+{\text{γ}}_{\text{l}}+{\text{βγ}}_{\text{kl}}+{\text{αβ}}_{\text{ik}}+{\text{αγ}}_{\text{il}}+{\text{αβγ}}_{\text{ikl}}+{\text{ε}}_{\text{ijkl}}$$

where y_ijkl_ = measured variable; μ = overall mean; α_i_ = year effect (i = 1,2); ρ_j(i)_ = blocks (j = 1,…,4) nested within the year; β_k_ = wheat (k = 1,…,12); γ_l_ = faba bean (l = 1,2); βγ_kl_ = wheat × faba bean interaction; αβ_ik_ = year × wheat interaction; αγ_il_ = year × faba bean interaction; αβγ_ikl_ = year × wheat × faba bean interaction; and ε_ijkl_ = residual error.

For the faba bean yield in mixed cropping, the 2019 and 2020 data were separately analyzed using the following ANOVA model:


(Model3) $${\text{y}}_{\text{ijk}}=\text{μ}+{\text{ρ}}_{\text{i}}+{\text{α}}_{\text{j}}+{\text{β}}_{\text{k}}+{\text{αβ}}_{\text{jk}}+{\text{ε}}_{\text{ijk}}$$

where y_ijk_ = faba bean grain yield; μ = overall mean; ρ_i_ = block effect (i = 1,…,4); α_j_ = wheat(*j* = 1,…, 12);β_k_ = faba bean (k = 1,2); αβ_jk_ = wheat × faba bean interaction; and ε_ijk_ = residual error.

For durum wheat grain protein content, the data from 2019 and 2020 were separately analyzed because of the highly significant heteroscedasticity of residual errors. The cropping system, including pure and mixed crop combinations with the two faba bean varieties, was included as the main effect in the following ANOVA model:


(Model4) $${\text{y}}_{\text{ijk}}=\text{μ}+{\text{ρ}}_{\text{i}}+{\text{α}}_{\text{j}}+{\text{β}}_{\text{k}}+{\text{αβ}}_{\text{jk}}+{\text{ε}}_{\text{ijk}}$$

where y_ijk_ = wheat grain protein content; μ = overall mean; ρ_i_ = block effect (i = 1,…,4); α_j_ = wheat(*i* = 1,…, 12); β_k_ = cropping system (k = 1,2,3: wheat pure cropping and mixed cropping with two faba bean varieties); αβ_jk_ = wheat × cropping system interaction; and ε_ijk_ = residual error.

Tukey’s honestly significant difference (HSD) test was applied for multiple comparisons among means for the main effects, and pairwise comparisons (with Bonferroni correction) were used for interactions. Moreover, confidence intervals were calculated to test significant differences from one of the mean LER_total_ values.

### Multivariate Analysis

To obtain a comprehensive picture of the effectiveness of mixed cropping, a multivariate approach was applied based on a combined analysis of the most representative variables describing the overall performance of mixed crop combinations: total yield, LER_total_, and ln(LER_ratio_). Specifically, principal component analysis (PCA) was performed using Pearson’s correlation matrix (Rencher, 2002), followed by cluster analysis (CA; Euclidean distance and UPGMA clustering), to identify the possible patterns of mixed crop combinations on the PCA scatterplot. For multivariate analysis, data from both years were combined in a single data file, with each combination of mixed crop and year considered as operational taxonomic units (OTUs) (Sneath and Sokal, 1973).

### Faba Bean Variety Rumbo

Data of the variety Rumbo were evaluated only in 2019, and the results of univariate analysis and PCA are summarized in Supplementary Material.

## Results

### Pure Cropping

All sources of variation (ANOVA, Model 1) were significant. As expected, mean grain yield across years was significantly higher for most durum wheat varieties than for faba bean varieties (Table 1A), with a highly significant positive correlation between years (*r* = 0.84, *P* < 0.01). Claudio was the best performing durum wheat variety, and its grain yield was significantly higher than that of most other varieties; Aureo was the lowest yielding variety. Pairwise contrasts (Table 1B) showed that the significant pure crop × year interaction was mainly due to the lower yield of both faba bean varieties in 2020 than in 2019 (*P* < 0.001). Only Nazareno and Aureo produced significantly (*P* < 0.05) higher mean yield in 2020 than in 2019. Therefore, in pure cropping, faba bean varieties were more influenced by the growing season than durum wheat varieties, reflecting the well-known yield instability of faba bean (Flores et al., 1996).

**TABLE 1:** Pure crop yields (Mg ha^–1^).

Varieties	(A) Pure crop^1^	(B) Pure crop × Year		
		2019	2020	P^2^
***Durum wheat***				
Claudio	6.77^a^	6.60	6.93	
Antalis	6.20^ab^	6.18	6.21	
Marco Aurelio	6.03^ab^	5.77	6.29	
Nazareno	6.00^ab^	5.43	6.58	*
Achille	6.00^ab^	6.15	5.85	
Odisseo	5.68^b^	5.72	5.65	
Natur	5.39^b*c*^	4.94	5.83	
Rangodur	5.35^b*c*^	4.94	5.75	
Tirex	5.32^b*c*^	4.88	5.76	
Svevo	5.31^b*c*^	5.15	5.46	
SanCarlo	4.61^cd^	4.38	4.84	
Aureo	3.99*^d^*	3.39	4.58	*
***Faba bean***				
Chiaro di Torrelama	4.27^d^	5.05	3.50	***
Prothabat69	3.93^d^	4.52	3.33	***

*(A) Pure crop main effect: multiple comparisons of mean yield across years (HSD test). (B) Pure crop × Year interaction: contrasts (with Bonferroni correction) performed separately for each variety between 2019 and 2020.*

*^1^Difference in means followed by different letters are statistically significant (P < 0.05).*

^2^P-value: contrasts between means followed by * or *** are significant at P < 0.05 or P < 0.001, respectively.

### Mixed Cropping

#### Durum Wheat Grain Yield

**FIGURE 1:**
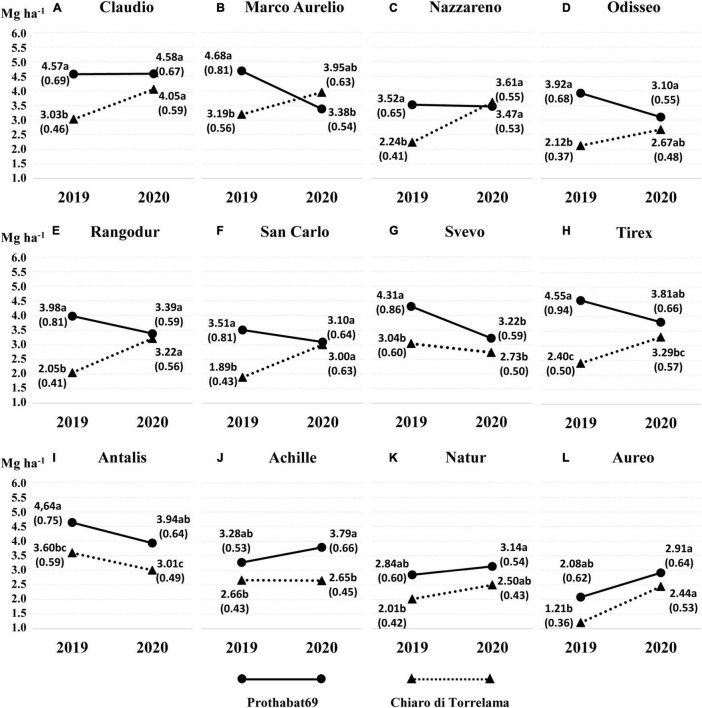
Grain yield in 2019 and 2020 of each durum wheat variety **(A–L)** in mixed cropping with two faba bean varieties (Chiaro di Torrelama and Prothabat69). Means followed by different letters are significantly different (*P* < 0.05). In parenthesis, the land equivalent ratio (LER) of wheat (LER_w)_ values are reported.

The year main effect (*P* = 0.13) and the wheat × faba bean interaction (*P* = 0.07) were not significant (ANOVA, Model 2). All the remaining sources of variation were highly significant (*P* < 0.001). Therefore, particular attention was paid to the second-order interaction (wheat × faba bean × year) and Figure 1 summarizes the performance of durum wheat varieties in mixed cropping with the two faba bean varieties.

Eight durum wheat varieties (Figures 1A–H) were characterized by a significantly higher yield with Prothabat69 than with Chiaro di Torrelama in 2019, whereas no significant differences were observed in 2020. Therefore, in 2019, these varieties highlighted a significantly lower performance in mixed cropping with Chiaro di Torrelama than with Prothabat69, as also reflected by the respective LER_w_ values. Significantly higher yields in combination with Prothabat69 than Chiaro di Torrelama were recorded for Antalis in both years (Figure 1I) and Achille in 2020 (Figure 1J), while the performance of Natur was relatively stable across years (Figure 1K). Aureo and Nazareno were characterized by a significant increase in yield from 2019 to 2020 in pure cropping (Table 1); in mixed cropping, however, this trend was detected only in the combination with Chiaro di Torrelama (Figures 1C,L). Moreover, pure crop yield was not correlated with LERw in both years.

Overall, the results of grain yield and LER_w_ highlighted a wide range of responses of the 12 durum wheat varieties in mixed cropping with the two varieties of faba bean. Performance as pure and mixed crops was also differently affected by the year of cultivation.

#### Faba Bean Yield

The statistical analysis of faba bean yield in mixed cropping was performed separately for each year because of the highly significant heteroscedasticity of residual errors. ANOVA (model 3) revealed significant main effects, but it was only in 2020 that the wheat × faba bean interaction was significant.

The mean yield of Chiaro di Torrelama was significantly higher than that of Prothabat69 both in 2019 (3.38 vs. 2.14 Mg ha^–1^) and 2020 (1.58 vs. 1.38 Mg ha^–1^), suggesting different general mixing abilities of the two faba bean varieties. Moreover, the performance of both faba bean varieties was poorer in 2020 than in 2019, reflecting the same trends observed in pure cropping. Regarding the main effect of durum wheat varieties on faba bean yield, in 2019, mixed cropping with four varieties, namely Nazareno, Natur, Achille, and Odisseo, showed significantly higher faba bean yields than most other combinations (Table 2A).

**TABLE 2:** Faba bean yield (Mg ha^–1^).

Durum wheat varieties	(A) Faba bean yield (Mg ha^–1^) in 2019			
	Main factor^1^	Wheat × Faba bean		
		ChTL^2^	Pr69^2^	P^3^
Nazareno	3.28^a^	3.95 (0.78)	2.62 (0.58)	***
Natur	3.24^a^	4.01 (0.79)	2.47 (0.55)	***
Achille	3.09^a^	3.56 (0.71)	2.61 (0.58)	**
Odisseo	3.06^a^	3.54 (0.70)	2.59 (0.57)	**
Rangodur	2.94^ab^	3.72 (0.74)	2.16 (0.48)	***
Aureo	2.89^abc^	3.52 (0.70)	2.25 (0.50)	***
SanCarlo	2.79^abc^	3.58 (0.71)	2.01 (0.44)	***
Marco Aurelio	2.43^bc^	3.11 (0.62)	1.75 (0.39)	***
Tirex	2.40^bc^	2.97 (0.59)	1.83 (0.40)	***
Antalis	2.35^bc^	2.77 (0.55)	1.93 (0.42)	*
Claudio	2.35^bc^	3.07 (0.61)	1.63 (0.36)	***
Svevo	2.27^c^	2.74 (0.54)	1.80 (0.40)	**

**Durum wheat varieties**	**(B) Faba bean yield (Mg ha** ^–^ **^1^) in 2020**			
	**Main factor^1^**	**Wheat × Faba bean**		
		**ChTL^2^**	**Pr69^2^**	**P^3^**

Natur	1.74^a^	1.78 (0.51)	1.71 (0.51)	
Achille	1.64^ab^	1.90 (0.55)	1.37 (0.41)	***
Antalis	1.55^abc^	1.71 (0.49)	1.40 (0.42)	
Rangodur	1.51^abc^	1.56 (0.45)	1.47 (0.44)	
Claudio	1.50^abc^	1.54 (0.44)	1.46 (0.44)	
Marco Aurelio	1.50^abc^	1.49 (0.43)	1.51 (0.45)	
Nazareno	1.44^bc^	1.50 (0.43)	1.37 (0.41)	
Odisseo	1.43^bc^	1.41 (0.41)	1.44 (0.44)	
Svevo	1.39^bc^	1.54 (0.44)	1.25 (0.37)	
SanCarlo	1.38^bc^	1.33 (0.38)	1.43 (0.43)	
Aureo	1.36^c^	1.80 (0.52)	0.92 (0.28)	***
Tirex	1.31^c^	1.40 (0.40)	1.21 (0.36)	

*Results of 2019 (A) and 2020 (B) field trials, including multiple comparisons (HSD test) among the mean yield of faba bean varieties (wheat as the main factor) and contrasts between mean yields of the two faba bean varieties within each mixed crop combination (wheat × faba bean interaction). Land equivalent ratio (LER) for faba bean (LER_fb)_ values are shown in parenthesis.*

*^1^Means followed by different letters are significantly different (P < 0.05).*

*^2^Variety name abbreviations: ChTL, Chiaro di Torrelama; Pr69, Prothabat69.*

^3^P-value: contrasts between means followed by *, **, or *** are statistically significant at P < 0.05, P < 0.01, and P < 0.001, respectively.

In 2019, there was a highly significant and negative correlation (*r* = −0.72, *P* < 0.01) between durum wheat and faba bean yield, suggesting that the average performance of faba bean significantly varied according to the durum wheat variety included as a combination crop. Therefore, the average yield of faba bean has increased as the yield of wheat decreased, suggesting some level of balance due to the interaction between the two crops in mixed cropping. In contrast, in 2020, no correlation between durum wheat and faba bean yield was detected, as a consequence of the lower average performance of faba bean. Interestingly, the mean faba bean yield in mixed cropping with Natur was the highest in both years, suggesting that Natur could be considered as the least competitive variety for faba bean in the set of durum wheat varieties evaluated.

Although in 2020 the wheat × faba bean interaction was significant, Chiaro di Torrelama performed better than Prothabat69 only in mixed cropping combination with Achille and Aureo (Table 2B).

Overall, faba bean was more affected by the different growing seasons than durum wheat, and the response of both faba bean and durum wheat was closely related to the companion variety included in mixed cropping.

#### Total Yield and LER_total_

The main effects of durum wheat and faba bean, as well as the durum wheat × faba bean × year interaction, were significant. Total yields of mixed crops including Claudio, Marco Aurelio, and Antalis, averaged across faba bean varieties and years, were significantly higher than those from most other combinations, whereas the mean total yield of mixed crops including Aureo was the lowest (Table 3A). Therefore, high variability due to the average effect of durum wheat varieties on the total yield of mixed crops was detected.

**TABLE 3:** Total yield and LER_total_.

Durum wheat varieties	Total yield (Mg ha^–1^)^1,2^								
	(A) Overall Mean	(B) Durum wheat × Faba bean × Year				(C) LER_total_^3^			
		2019		2020		2019		2020	
		ChTL^4^	Pr69^4^	ChTL^4^	Pr69^4^	ChTL^4^	Pr69^4^	ChTL^4^	Pr69^4^
Claudio	5.98^a^	6.09^a^	6.20^a^	5.59^a^	6.04^a^	1.07	1.05	1.03	1.11*
Marco Aurelio	5.76^ab^	6.30^a^	6.43^a^	5.43^ab^	4.88^b^	1.18**	1.20**	1.05	0.99
Antalis	5.74^ab^	6.37^a^	6.56^a^	4.71^b^	5.33^b^	1.14**	1.18**	0.98	1.06
Nazareno	5.57^abc^	6.18^a^	6.14^a^	5.11^b^	4.84^b^	1.20**	1.24**	0.98	0.94
Achille	5.46^bc^	6.23^a^	5.89^ab^	4.55^c^	5.15^bc^	1.15**	1.11*	1.00	1.06
Rangodur	5.38^bcd^	5.76^ab^	6.14^a^	4.77^b^	4.85^b^	1.15**	1.29**	1.01	1.03
Tirex	5.36^bcd^	5.37 ^b^	6.38^a^	4.69^b^	5.01^b^	1.09	1.34**	0.98	1.02
Odisseo	5.20^cd^	5.67^a^	6.51^a^	4.09^b^	4.54^b^	1.07	1.26**	0.88*	0.98
Svevo	5.16^cd^	5.78^a^	6.11^a^	4.27^b^	4.47^b^	1.14**	1.26**	0.94	0.97
Natur	5.11^cd^	6.02^a^	5.30^ab^	4.27^c^	4.84^bc^	1.22**	1.14**	0.94	1.05
San Carlo	4.96^d^	5.47^a^	5.52^a^	4.33^b^	4.53^ab^	1.14**	1.25**	1.01	1.07
Aureo	4.28*^e^*	4.73^a^	4.33^a^	4.24^a^	3.83^a^	1.06	1.11*	1.05	0.92

*(A) Multiple comparison (HSD test) of the mean total yields of mixed crops, averaged across faba bean varieties and years. (B) Second-order interaction: pairwise contrasts between mean total yields of mixed crop combinations within each durum wheat variety. (C) LER_total_ values.*

*^1^Overall mean yield: differences in means followed by different letters are significant (P < 0.05).*

*^2^Pairwise contrasts (by rows) of mixed crop combinations for each durum wheat variety: differences in means followed by different letters are significant (P < 0.05).*

^3^LER_total_ values are significantly higher or lower than one at P < 0.05 (*) or P < 0.01 (**).

*^4^Variety name abbreviations: ChTL, Chiaro di Torrelama; Pr69, Prothabat69.*

The mean total yield of mixed crops including Prothabat69 (5.41 Mg ha^–1^) was significantly higher than that of mixed crops including Chiaro di Torrelama (5.25 Mg ha^–1^). Therefore, including the faba bean variety with the lowest performance in mixed cropping resulted in a higher mean total yield because of the increased performance of durum wheat.

Regarding the wheat × faba bean × year interaction, pairwise contrasts (Table 3B) revealed that the total yields of mixed crops including Claudio and Aureo were the most stable across faba bean varieties and years, whereas for mixed cropping including Antalis, Nazareno, Odisseo, and Svevo the total yield was significantly higher in 2019 than in 2020 for combinations with both faba bean varieties. In contrast, mixed cropping including Marco Aurelio, Rangodur, and Tirex produced a significantly lower total yield in 2020 only in combination with Prothabat69, whereas Achille, Natur, and San Carlo produced lower yields in 2020 in combination with Chiaro di Torrelama. Interestingly, comparisons within the year highlighted no significant differences in total yield among the varieties, except for Tirex in 2019.

Moreover, in 2019, LER_total_ values were significantly higher than one for almost all mixed crop combinations, except for Claudio (Table 3C). Meanwhile, in 2020, the LER_total_ values for almost all mixed crop combinations were not significantly different from one, except the Claudio–Prothabat69 and Odisseo–Chiaro di Torrelama combinations, which showed LER_total_ values significantly higher and lower than one, respectively.

Overall, a trend toward convergence to similar overall performance in terms of total yield and LER_total_ was noted for the mixed crop combinations of each durum wheat variety, together with different responses between years. However, the total yield and LER_total_ did not provide information on the relationship between the relative performances of the varieties of the two crops, as expressed by LER_w_ and LER_fb_, the values of each mixed crop combination. For this purpose, the LER_ratio_ was analyzed following the ln- transformation.

#### ln(LER_ratio_)

The ANOVA (model 2) revealed highly significant wheat and faba bean main effects and significant wheat × faba bean × year interactions (*P* < 0.001). Regarding the average effects of the two faba bean varieties, the mean ln(LER_ratio_) values were positive (0.410) and negative (−0.112) for Prothabat69 and Chiaro di Torrelama, respectively, and the difference was highly significant. Therefore, the average relative performance of the durum wheat was significantly better than faba bean in mixed cropping with Prothabat69, whereas Chiaro di Torrelama showed, on average, a better performance than durum wheat in mixed cropping.

The results of multiple comparisons among overall mean ln(LER_ratio_) values of the durum wheat varieties are presented in Table 4A. There were significant differences between two groups of varieties, characterized by positive (from 0.262 to 0.411) and negative (from −0.183 to −0.006) ln(LER_ratio_) values. Three varieties, namely San Carlo, Rangodur, and Aureo, were ranked as intermediate, while Tirex (the highest) and Natur (the lowest) showed significantly different extreme means.

**TABLE 4:** ln(LER_ratio_).

Durum wheat varieties	(A) Main factor		(B) Durum wheat × faba bean × year interaction					
	Durum wheat		ln(LER_ratio_) in 2019			ln(LER_ratio_) in 2020		
	Mean^1^	Ratio	ChTL^2^	PR69^2^	P^3^	ChTL^2^	PR69^2^	P^3^
Tirex	0.411^a^	(1.51:1)	−**0.168** (1:1.18)	0.851 (2.34:1)	***	0.358 (1.43:1)	0.602 (1.83:1)	
Svevo	0.354^ab^	(1.42:1)	0.094 (1.10:1)	0.748 (2.11:1)	**	0.121 (1.13:1)	0.454 (1.57:1)	
Marco Aurelio	0.301^ab^	(1.35:1)	−**0.099** (1:1.10)	0.746 (2.11:1)	***	0.382 (1.47:1)	0.177 (1.19:1)	
Claudio	0.277^ab^	(1.32:1)	−**0.270** (1:1.31)	0.679 (1.97:1)	***	0.287 (1.33:1)	0.412 (1.51:1)	
Antalis	0.262^ab^	(1.30:1)	0.063 (1.06:1)	0.579 (1.78:1)		−**0.010 (1:1.01)**	0.418 (1.52:1)	
San Carlo	0.255^abc^	(1.29:1)	−**0.501 (1:1.65)**	0.624 (1.86:1)	***	0.495 (1.64:1)	0.402 (1.50:1)	
Rangodur	0.114^bcd^	(1.12:1)	−**0.583 (1:1.79)**	0.525 (1.69:1)	***	0.224 (1.25:1)	0.289 (1.36:1)	
Aureo	0.104^bcd^	(1.11:1)	−**0.686 (1:1.99)**	0.234 (1.26:1)	***	0.038 (1.04:1)	0.829 (2.29:1)	***
Nazareno	**−0.006^cde^**	**(1:1.01)**	−**0.635 (1:1.89)**	0.121 (1.12:1)	***	0.233 (1.26:1)	0.259 (1.30:1)	
Odisseo	**−0.022^cde^**	**(1:1.02)**	−**0.648 (1:1.91)**	0.174 (1.19:1)	***	0.156 (1.17:1)	0.228 (1.26:1)	
Achille	**−0.076^de^**	**(1:1.08)**	−**0.498 (1:1.65)**	−**0.085 (1:1.09)**		−**0.184 (1:1.20)**	0.464 (1.59:2)	***
Natur	**−0.183^e^**	**(1:1.20)**	−**0.678 (1:1.97)**	0.071 (1.07:1)	***	−**0.172 (1:1.19)**	0.046 (1.05:1)	

*(A) Multiple comparisons (HSD test, P < 0.05) of overall means across faba bean varieties and years. (B) Second-order interaction: pairwise contrasts (with Bonferroni correction) performed within the year between mixed crops of each durum wheat variety. For each ln(LER_ratio_) mean, the corresponding LER_w_/LER_fb_ is shown in parenthesis; negative values are boldfaced.*

*^1^Means followed by different letters are significantly different (P < 0.05).*

*^2^Variety name abbreviations: ChTL, Chiaro di Torrelama; Pr69, Prothabat69.*

^3^Differences in means are significant at **P < 0.01 or ***P < 0.001.

Regarding the two-way interaction (Table 4B), in 2019, the mean ln(LER_ratio_) values were negative and positive for almost all mixed crop combinations including Chiaro di Torrelama and Prothabat69, respectively. The negative ln(LER_ratio_) values for Chiaro di Torrelama indicate that in 2019, LER_fb_ was higher than LER_w_, whereas a contrasting trend was noted for Prothabat69. Therefore, in 2019, Chiaro di Torrelama showed a better performance than Prothabat69 in mixed cropping with most durum wheat varieties. However, the performance of Chiaro di Torrelama in 2020 was much poorer than that in 2019. Consequently, the mean ln(LER_ratio_) values were positive for most mixed crop combinations with both faba bean varieties (Table 4B). Only Achille and Natur showed negative ln(LER_ratio_) values with Chiaro di Torrelama in 2020, suggesting that in less favorable growing seasons for faba bean, these two durum wheat varieties were characterized by the lowest competitive ability against the best performing faba bean variety.

Overall, the analysis of ln(LER_ratio_) values confirmed that the relative performance of cereal and legume crops is an important parameter when assessing the effectiveness of durum wheat–faba bean mixed cropping.

#### Durum Wheat Grain Protein Content

In both years, durum wheat grain protein content showed highly significant variances for cropping system and durum wheat × cropping system interaction (ANOVA, model 4). Multiple comparisons among cropping systems revealed that in both years, on average, mixed cropping increased the protein content of durum wheat (Table 5A), and the two faba bean varieties significantly differed in terms of their overall effect on this durum wheat quality trait. Regarding the wheat × cropping system interaction (Table 5B), most durum wheat varieties showed a significant increase in grain protein content in mixed cropping with Chiaro di Torrelama in both years, whereas little differences between mixed cropping with Prothabat69 and pure crops were detected in 2019.

**TABLE 5:** Durum wheat grain protein content (%) in 2019 and 2020.

	Grain protein (%)^3^					
	2019			2020		
	Pure	ChTL^3^	Pr69^3^	Pure	ChTL^3^	Pr69^3^
**(A) CS^1^**	16.0^c^	17.2^a^	16.6^b^	13.8^c^	14.8^a^	14.5^b^
**(B) Variety^2^**						
Achille	14.9	15.5	14.6	13.0	13.7	13.2
Antalis	14.4	15.1	14.9	12.8	13.7**	13.7**
Aureo	19.6	20.6*	20.4	15.5	17.5***	16.5***
Claudio	15.7	16.4	15.6	13.8	14.5*	14.4
Marco Aurelio	16.5	17.8**	17.4	14.7	15.6**	15.2
Natur	16.15	16.7	16.4	13.2	14.4***	14.4***
Nazareno	15.8	16.9**	16.2	14.4	14.8	14.8
Odisseo	14.9	17.1***	15.4	13.5	14.3**	14.1
Rangodur	16.1	16.7	16.8	13.1	14.3***	13.9**
SanCarlo	16.5	18.2***	17.1	14.1	15.4***	15.4***
Svevo	16.8	18.1***	17.6	14.2	15.0**	14.8
Tirex	15.1	17.6***	16.6***	12.9	14.2***	14.2***

*(A) Cropping system (CS) as the main factor: multiple comparisons among overall means of 12 durum wheat varieties in pure and mixed cropping systems within each year. (B) Wheat × Cropping system interaction: pairwise contrasts, between the mean of pure crop and each mixed crop combination for each durum wheat variety.*

*^1^Within a year, means followed by different letters are significantly different (HSD test, P < 0.05).*

^2^Within a year, the mean of each mixed crop is significantly different from that of the respective pure crop at *P < 0.05, **P < 0.01, or ***P < 0.001.

*^3^Faba bean variety name abbreviations: ChTL, Chiaro di Torrelama; Pr69, Prothabat69.*

#### Comprehensive Mixed Crop Performance

**FIGURE 2:**
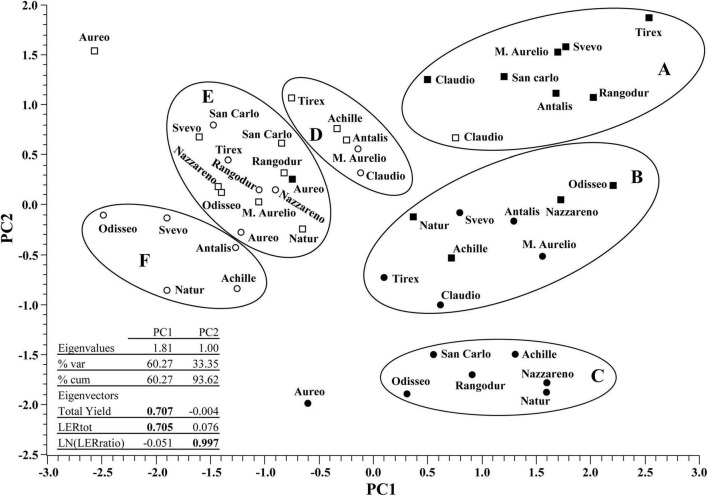
Scatterplot of principal component analysis (PCA). Circles and squares represent mixed crop combinations with Chiaro di Torrelama and Prothabat69, respectively. Mixed crops grown in 2019 and 2020 are indicated in black and white, respectively. Capital letters **(A–F)** indicate groups identified in cluster analysis (as shown in Supplementary Figure 2).

To further analyze the information obtained through univariate analyses, a more comprehensive approach based on PCA, followed by CA, was applied. For this analysis, the most important variables, summarizing different features of mixed cropping performance, were considered: total yield, LER_total_, and ln(LER_ratio_). The combined results of the PCA and CA, including eigenvalues and eigenvectors, are presented in Figure 2. The dendrogram obtained through CA is shown in Supplementary Figure 2.

The first two principal components (PC1 and PC2) were highly significant (Bartlett test, *P* < 0.001) and explained 93.6% of the total variation. PC1 was related to the total yield and LER_total_, and both variables showed positive PC1 eigenvector coefficients. Therefore, the higher the PC1 score, the higher the overall mixed crop performance as a combination of the total yield and LER_total_. Moreover, PC1 effectively highlighted the different performances of mixed crops in the 2 years; the PC1 scores were mainly positive and negative for the mixed crops evaluated in 2019 and 2020, respectively.

The ln(LER_ratio_) was not correlated with either total yield (*r* = −0.06, ns) or LER_total_ (*r* = 0.003, ns), but it was important for PC2, explaining 33.35% of the total variance. For PC2, high positive, intermediate, and low negative scores were related to mixed crops with a better performance of durum wheat than of faba bean, a similar performance of the two crops, and better performance of faba bean than of durum wheat, respectively.

The effectiveness of ln(LER_ratio_) in the characterization of mixed crop combinations was further confirmed using CA, which identified main clusters of mixed crops within each year, with only a few exceptions (Figure 2 and Supplementary Figure 2). In 2019, three main clusters (A, B, and C) with positive PC1 scores but different PC2 scores were detected. Only Claudio–Prothabat69, evaluated in 2020, was included in cluster A. Clusters A, B, and C were primarily discriminated against based on PC2 scores because the range of variation in PC1 scores was rather similar among the three clusters.

Cluster A included mixed crop combinations characterized by having only Prothabat69 as the faba bean companion, and ln(LER_ratio_) values were always positive, ranging between 0.41 (Claudio–Prothabat69 in 2019, LER_w_ = 1.51 × LER_fb_) and 0.85 (Tirex-Prothabat69; LER_w_ = 2.34 × LER_fb_). These results indicate that in the mixed crop combinations of cluster A, durum wheat varieties overcame Prothabat69 in relative performance, and all mixed crop combinations, except the one including Claudio in 2019 (LER_total_ = 1.05), showed LER_total_ values significantly higher than one. Interestingly, based on CA, the mixed crop combinations of Claudio–Prothabat69 evaluated in 2019 and 2020 formed a sub-cluster within cluster A (Supplementary Figure 2), with the lowest PC1 scores.

Cluster B showed intermediate PC2 scores. The lowest and highest PC2 scores were recorded for Claudio–Chiaro di Torrelama and Odisseo–Prothabat69, with ln(LER_ratio_) values of −0.27 (LER_w_ = 0.76 × LER_fb_) and 0.17 (LER_w_ = 1.18 × LER_fb_), respectively. Therefore, cluster B included mixed crop combinations that showed a more balanced LER_w_/LER_fb_ ratio in 2019.

Cluster C was characterized by mixed crop combinations, including only Chiaro di Torrelama, and showed low negative PC2 scores because the relative performance of the faba bean variety was higher than the durum wheat varieties. The ln(LER_ratio_) values ranged between −0.68 (Natur–Chiaro di Torrelama; LER_w_ = 0.51 × LER_fb_) and −0.50 (San Carlo–Chiaro di Torrelama; LER_w_ = 0.61 × LER_fb_).

The variability for PC2 scores was lower in 2020 than in 2019, as shown by the range of PC2 scores in the bi-dimensional scatterplot, and three main clusters (D, E, and F) were identified by CA (Figure 2). The PC1 scores in 2020 were lower than those in 2019 and the narrower range of variation in the PC2 scores reflected the lower performance of faba bean varieties in 2020 than in 2019.

The main difference between clusters D and E was related to the total yield, which was mostly higher and lower than 5 Mg ha^–1^, respectively, and their LER_total_ values were not significantly different from 1. Interestingly, cluster F showed negative PC2 scores and, similar to cluster C, included mixed crops combinations with only Chiaro di Torrelama as the faba bean companion crop.

Finally, the durum wheat variety Aureo warrants specific attention. In fact, PCA revealed that this variety acted as an outgroup in 2019 when cultivated in combination with Chiaro di Torrelama as well as in 2020 when cultivated in combination with Prothabat69. Aureo is a well-known low-yielding but high-quality variety. As a mixed crop, Aureo showed the poorest performance with both faba bean varieties in 2019 and with Prothabat69 in 2020. Its PC1 scores were low and, based on the PC2 score, this variety showed a better competitive ability against Prothabat69 than against Chiaro di Torrelama in both years. These results suggest that Aureo should be selected as a mixed crop with caution, as its performance is more closely linked to the companion faba bean variety and growing season than the other durum wheat varieties.

#### Results Including the Variety Rumbo

The faba bean variety Rumbo was included in 2019 alone and results regarding this variety are summarized as Supplementary Table 2 and Supplementary Figure 3 (principal component analysis with Rumbo). Mixed crops including Rumbo showed a wider range of PC1 scores and intermediate PC2 scores when compared to 2019 results of the other two faba bean varieties. Therefore, although limited to one trial, these results confirm that an interesting variability in mixed cropping characterized the set of faba bean varieties evaluated.

## Discussion

The present study examined the durum wheat–faba bean mixed cropping system using a set of varieties that are commonly cultivated in central Italy as pure crops. Two years of field trials provided information that could be usefully extended to future breeding for mixed cropping.

Durum wheat is a very important cereal in Italy and the Mediterranean Basin, this crop has been subjected to intense breeding, a wide range of varieties is currently available for farmers (De Vita et al., 2007), and as a pure crop, it ensures high and stable yields across environmental conditions. However, mixed cropping improves land-use efficiency, reduces the use of herbicides and nitrogen fertilizers, leading to more sustainable crop management than pure cropping. The results of the present study showed that by reducing N fertilization by 50%, mixed cropping resulted in a higher grain protein content.

Due to the good performance as a pure crop, farmers could not positively look at the replacement of pure durum wheat with mixed crops. Therefore, a proper and effective strategy, aimed at the valorization in the final products of the advantages related to agroecological and grain quality aspects, should be applied to make farmers choose to intercrop as an effective alternative practice to durum wheat pure crop. However, further studies are necessary on the effects of intercropping on other important durum wheat quality traits, such as yellowness and gluten index (Borrelli et al., 1999; De Vita et al., 2007; Magallanes-López et al., 2017).

In both years a highly significant correlation (*P* < 0.01) was found between durum wheat yield as pure and in mixed cropping, but pure crop yield was not correlated with LER_w_. Therefore, for the set of varieties evaluated here, the performance in pure crop could not be considered as an index of durum wheat performance in mixed cropping. For example, the variety Claudio ranked among the highest yielding ones in 2019 and 2020 both as pure and in mixed cropping. However, in 2019 the LER_total_ values of mixed crops including Claudio were the lowest. These results were due to its low LER_w_ value (LERw = 0.46) in combination with Chiaro di Torre Lama and its high LER_w_ value (LER_w_ = 0.69) with Prothabat69 that was followed by very low performance of this faba bean variety (LER_fb_ = 0.36). Differently, in 2020, both combinations including Claudio were the best-performing ones in terms of the LER_total_, because the high LER_w_ value compensated for the low general performance of faba bean. These results suggested that a specific relationship between the durum wheat and the faba bean variety, as also affected by environmental conditions, determined the overall performance of each mixed crop combination. This trend was further confirmed by the negative correlation found in 2019, followed by no correlation in 2020, between durum wheat and faba bean yields in mixed cropping.

Regarding faba bean, this crop is mainly used as feedstuff and plant breeding programs have been less extensively applied for this grain legume than for durum wheat, although it was traditionally a very important legume crop for animal feeding. Therefore, the range of faba bean varieties commercialized in Italy is restricted as compared to durum wheat, and the reintroduction of faba bean at the large scale is currently impeded by the instability of its performance over years, together with many socio-economic aspects, as highlighted by Brooker et al. (2015), Magrini et al. (2016), and Mamine and Farès (2020). Nevertheless, the differences in mixed cropping performance observed among the three faba bean varieties, including Rumbo that was evaluated only in 2019, suggested that genetic variability is available for breeders to select better faba bean genotypes for mixed cropping with durum wheat. The yield fluctuation of faba bean over years is also a very important constraint that hampered the spread of this grain legume in more rational crop rotations. Therefore, breeding to improve faba bean yield stability is of utmost importance to obtain high legume performance both in favorable and unfavorable growing seasons.

Interestingly, our results showed that the total yield of mixed crops was much higher than that of faba bean as a pure crop in both years, and farmers could also exploit the advantage of mixed cropping for better control of weeds as compared to faba bean pure cropping, due to the allelopathic effects of the cereal. For these reasons, farmers could, at present, look at mixed cropping as a valuable alternative to faba bean pure crops, especially because in unfavorable years for the legume crops, the yield of wheat would balance the lowered yield of the grain legume in the harvested mixed grain. Hopefully, plant breeding will identify new faba bean genotypes that will avoid the differences in the overall performance of durum wheat-faba bean mixed cropping observed comparing the results obtained in 2019 and 2020.

However, given the lack of genotypes selected for mixed cropping, at present, the implementation of durum wheat-faba bean mixed cropping in the transition toward more sustainable agricultural systems must rely on varieties available in the market and selected for sole cropping. Therefore, at present, mixed cropping could be considered a valid alternative to faba bean pure crops, whereas breeding efforts are requested to make mixed cropping more competitive than durum wheat pure crops for farmers.

Moreover, the LER_ratio_ highlighted that the relative performance of the cereal and the legume varied across different mixed crop combinations and over years. Therefore, in inbreeding programs, the LER_ratio_ could be a valuable parameter, together with selection for better stability across years, for the characterization of mixed crop combinations. As a matter of fact, in 2019 durum wheat performance was influenced by the faba bean variety included as a companion crop, because most durum wheat varieties were characterized by a significantly higher yield in combination with Prothabat69 than with Chiaro di Torrelama, and mixed crops including Rumbo showed an intermediate behavior. In 2020, most durum wheat varieties did not show a significant difference in grain yield between combinations with the two faba bean varieties, only Antalis and Achille, retaining a significantly higher yield with Prothabat69 than Chiaro di Torrelama. Therefore, in both years, the durum wheat-faba bean combination significantly affected the overall performance of the mixed crop as a whole, and the LER_ratio_ showed a high discriminant ability among the mixed crop combinations, reflecting an important aspect that would have otherwise been missed by considering only the total yield and LER_total_.

Overall, the univariate (ANOVA) and multivariate (PCA and CA) analyses provided complementary information for interpreting the performance of durum wheat–faba bean mixed cropping. The average performance of each variety in mixed cropping could provide information on the general mixing ability, but the negative correlation between durum wheat and faba bean yield in 2019 and the overall clustering of mixed crop combinations over years suggested that specific mixing ability should not be disregarded. Therefore, the multivariate analysis of grain yield, LER, and LER_ratio_ allowed the characterization of all mixed crop combinations through a comprehensive evaluation of their overall performance. Indeed, in 2019 all mixed crops of durum wheat varieties grouped in cluster A (high positive PC2 score) shifted to cluster B or C when Chiaro di Torrelama replaced Prothabat69 as a companion crop. Therefore, the relative performance of these durum wheat varieties decreased in combination with Chiaro di Torrelama. The same result was observed for durum wheat varieties that, in combination with Prothabat69, were included in cluster B but shifted to cluster C when Chiaro di Torrelama was the companion crop. Interestingly, this trend was retained also in 2020, although the range of variation of PC2 score was lower than that detected in 2019.

The multivariate approach highlighted the clear differentiation between mixed crops based on the combinations of durum wheat and the faba bean varieties. Therefore, the principal component scores could be applied as indices of selection within breeding programs aimed to simultaneously improve both cereal and legume performance. This approach could allow the identification of the best combinations that could be considered as an alternative to durum wheat pure crop, faba bean pure crop, or both. Of course, the interpretation of PC scores could vary based on the features of the genotypes of durum wheat and faba bean under evaluation.

## Conclusion

The present study involved durum wheat, which is probably the most important cereal in the Mediterranean area, and it is involved in a market that asks the farmers to combine high yield with high-quality parameters. All varieties included in the present study not only reflected these needs but also represented a good sample to test their mixing ability with the aim of gathering information on genetic variability that could be available for future breeding programs. The evaluation of this representative set of durum wheat varieties highlighted that efforts are needed to select new durum wheat genotypes because selection for pure crops did not reflect their performance in mixed cropping.

Moreover, although a restricted number of faba bean varieties was included, results of PCA and CA suggested that different combinations of durum wheat and faba bean varieties could result in different relative performances of the cereal and the legume crops. The faba bean choice deeply influenced the performance of durum wheat and, consequently, of the whole mixed crop combination, as better highlighted by the multivariate rather than univariate analysis. Therefore, the analysis at the mixed crop combination level is an important feature to be considered in further breeding programs for durum wheat-faba bean mixed cropping. However, breeding efforts for faba bean in mixed cropping must also be addressed to reduce the instability of performance over years. For this purpose, a wider range of faba bean varieties available in the EU Common Catalog of Plant Varieties, together with further germplasm accessions, could be evaluated and included in specifically targeted breeding programs for mixed cropping.

In conclusion, consideration is necessary about climate change. In the last years, agriculture has been facing the effects of strong year-to-year variation in environmental conditions that deeply influenced crop performance. The 2 years involved in the present study reflected the variability of crop performance under very contrasting growing seasons, confirming, based on the set of varieties available in the market, the much higher resilience of durum wheat than faba bean. Therefore, our results highlighted the need for a much more intense breeding work for faba bean than for durum wheat to let mixed cropping be more positively evaluated by farmers as an alternative to durum wheat pure crops. *Vice versa*, durum wheat-faba bean mixed cropping is a real opportunity as an alternative to faba bean pure cropping, especially in low input conventional or organic farming. However, the more constant performance of faba bean varieties in mixed cropping is requested, and therefore breeding programs for this grain legume should also involve multiyear trials together with selection carried out under mixed cropping with durum wheat genotypes.

## Data Availability Statement

The raw data supporting the conclusions of this article will be made available by the authors, without undue reservation.

## Author Contributions

Both authors listed have made a substantial, direct, and intellectual contribution to the work, and approved it for publication.

## Conflict of Interest

The authors declare that the research was conducted in the absence of any commercial or financial relationships that could be construed as a potential conflict of interest.

## Publisher’s Note

All claims expressed in this article are solely those of the authors and do not necessarily represent those of their affiliated organizations, or those of the publisher, the editors and the reviewers. Any product that may be evaluated in this article, or claim that may be made by its manufacturer, is not guaranteed or endorsed by the publisher.
