# Supplementary figures and images for: A Comprehensive Approach to Evaluate Durum Wheat–Faba Bean Mixed Crop Performance
Source: Front Plant Sci. 2022 Mar 23;13:733116. doi: 10.3389/fpls.2022.733116 (PMC8984478; doi:10.3389/fpls.2022.733116)

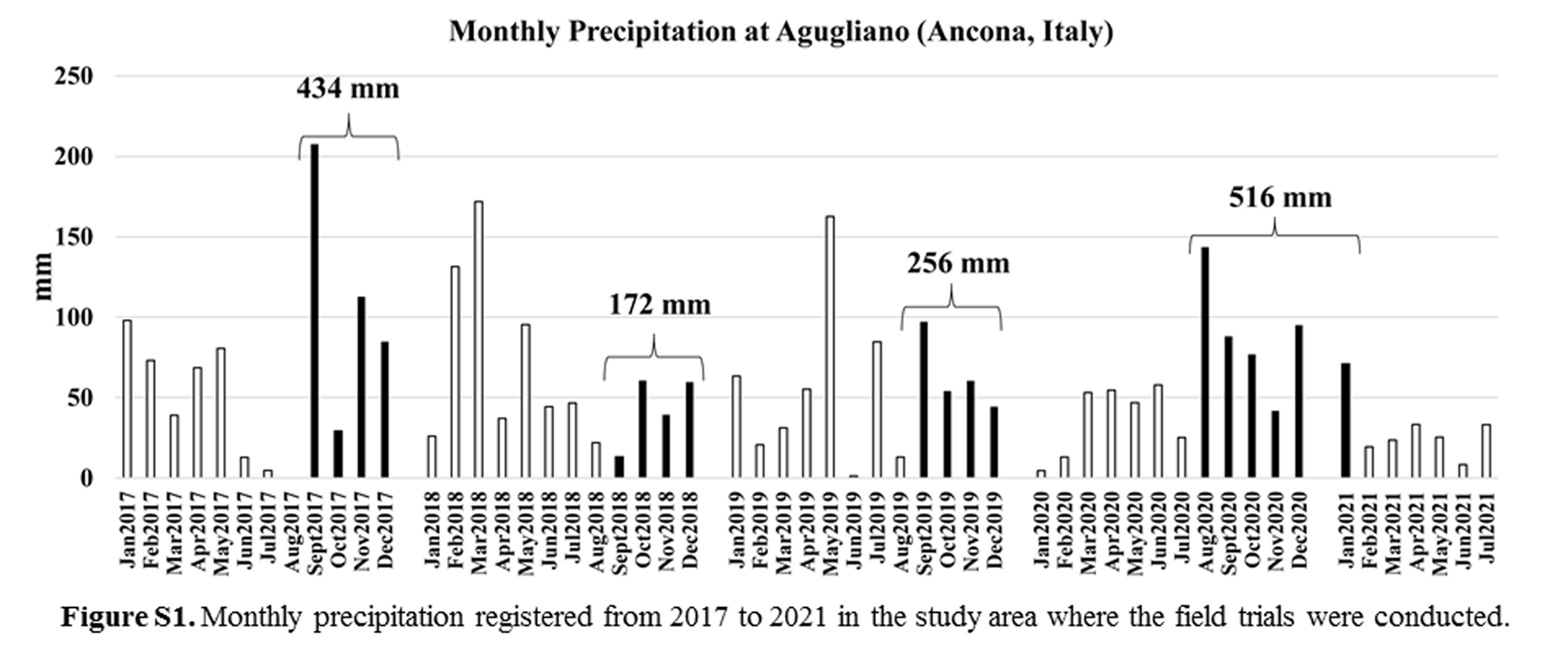

Supplement: Supplementary file 1 [file Image_1.tif]

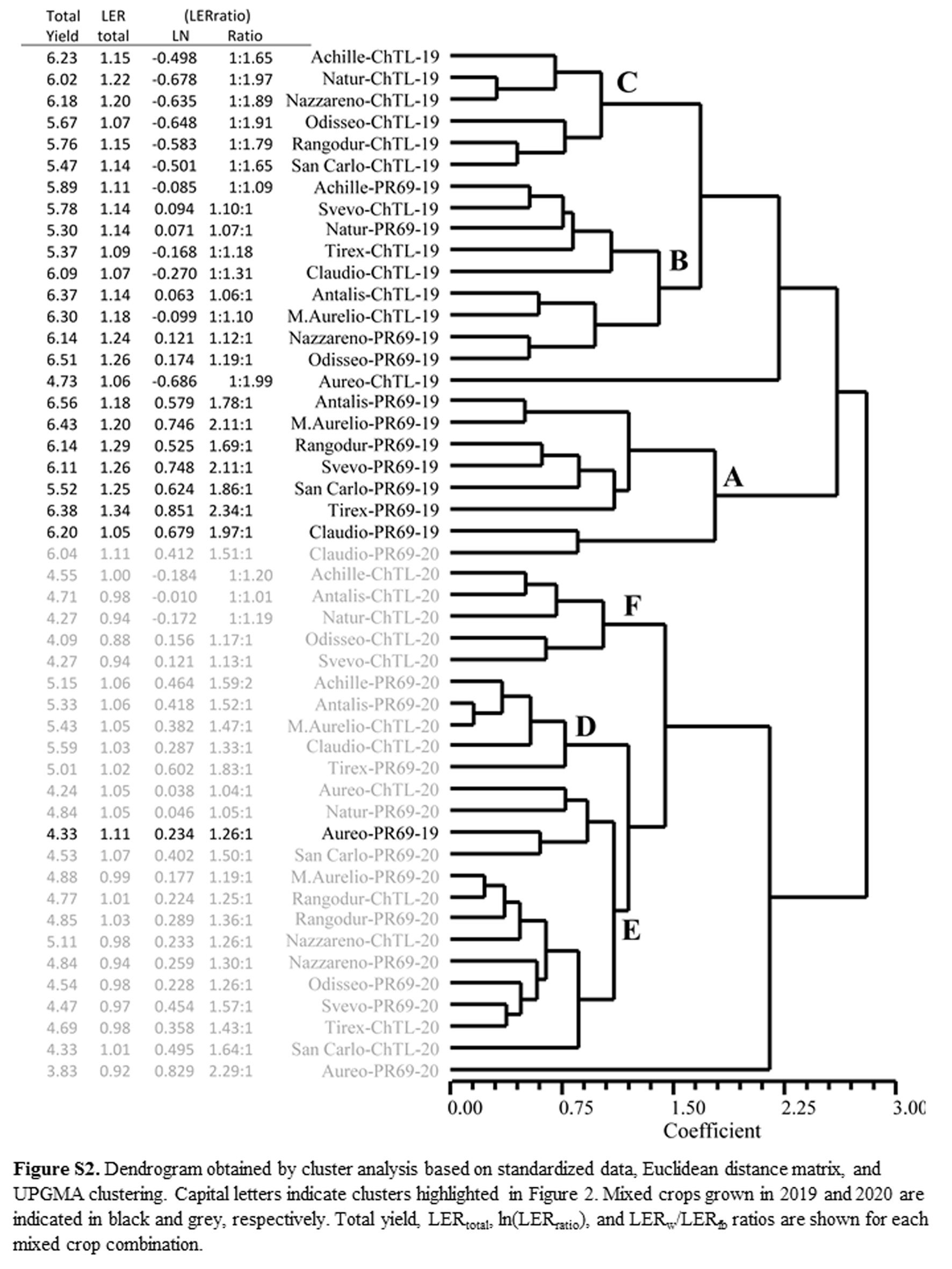

Supplement: Supplementary file 2 [file Image_2.tif]

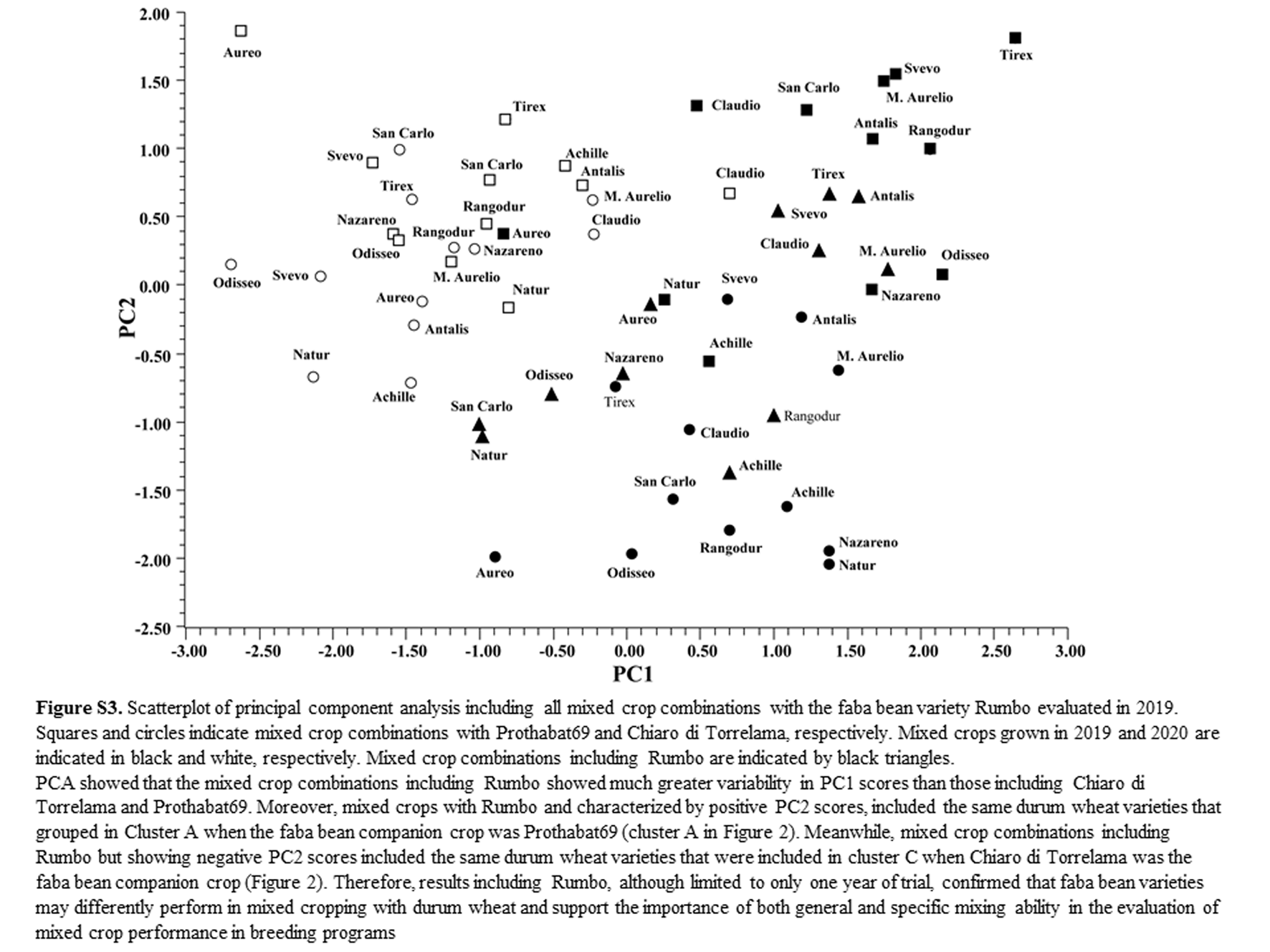

Supplement: Supplementary file 3 [file Image_3.tif]
