# Supplementary material for: A Comprehensive Approach to Evaluate Durum Wheat–Faba Bean Mixed Crop Performance
Source: Front Plant Sci. 2022 Mar 23;13:733116. doi: 10.3389/fpls.2022.733116 (PMC8984478; doi:10.3389/fpls.2022.733116)
Supplement: Supplementary file 4 [file Table_1.DOCX]

**Table S1.** Main characteristics of durum wheat and faba bean varieties included in the field trials.

| Durum wheat varieties | Release date | Breeder | Pedigree^1^ | Sowing time | Yield | Heading time | Thousand-kernel weight | Test weight (kg hl^-1^) | Protein content (%) |
| --- | --- | --- | --- | --- | --- | --- | --- | --- | --- |
| Achille | 2006 | Agroservice | L.37/05 × AG-4073 | Winter | High | Intermediate | >51 | High | 13.0–13.5 |
| Antalis | 2013 | Limagrain | n.a. | Winter | High | Intermediate | 51–53 | High | 12.0–13.5 |
| Aureo | 2009 | Società Produttori Sementi | Kofa/Svevo | Winter | Moderate–high | Early | >51 | Good | >14.5 |
| Claudio | 2006 | SIS | Sel.Cymmit35/Durango//IS1938/Grazia | Winter | Very high | Intermediate | 45–50 | High | High |
| Marco Aurelio | 2010 | SIS | Orobel//Arcobaleno/  Svevo | Winter | Moderate–high | Intermediate | 53–58 | Good | High |
| Natur | 2014 | RAGT | n.a. | Winter | High | Intermediate–late |  |  | 13.8 |
| Nazareno | 2016 | CGS | n.a. | Winter | High | Intermediate | 46–50 | High | High |
| Odisseo | 2011 | Società produttori Sementi | Svevo/le24 | Winter | Very high | Intermediate–late | >54 | High | >13.0 |
| Rangodur | 2015 | RAGT | n.a. | Winter | High | Intermediate | >40 | Modetate | >13.0 |
| SanCarlo | 1996 | Maliani genetica srl | Grazia × Degamit | Winter | Good | Intermediate | 55–60 | High | >14.0 |
| Svevo | 1995 | Società produttori Sementi | Line Cymmit/Zenit | Winter | Moderate–high | Very early | >51 | High | >14.0 |
| Tirex | 2007 | APSOV | Svevo ×Nefer | Winter | High | Early | 47–49 | High | High |
|  |  |  |  |  |  |  |  |  |  |
| **Faba bean varieties** | Release date | Breeder | Sowing time | Seed color | Thousand-seed weight | Protein content (%) |  |  |  |
| Chiaro di Torrelama | 1982 | Agroservice Spa | October–March | Light brown | 330–460 | 24–26 |  |  |  |
| Rumbo | n.a. | Semillas Fito | October–March | Light brown | 380–580 | 25–27 |  |  |  |
| Prothabat69 | n.a. | Semillas Battle | October–March | Light brown | 450–550 | 30–35 |  |  |  |

^1^n.a.=not available.
