# Supplementary material for: A Comprehensive Approach to Evaluate Durum Wheat–Faba Bean Mixed Crop Performance
Source: Front Plant Sci. 2022 Mar 23;13:733116. doi: 10.3389/fpls.2022.733116 (PMC8984478; doi:10.3389/fpls.2022.733116)
Supplement: Supplementary file 5 [file Table_2.DOCX]

Table S2. Overall results of the faba bean variety Rumbo, included only in the 2019 field trial.

| durum wheat variety | wheat yield Mg ha^-1^ | Rumbo^1^ yield Mg ha^-1^ | Total yield Mg ha^-1^ | LER_w_ | LER_fb_ | LER_total_ | ln(LER_ratio_) | LERratio | Wheat Protein% | Wheat Std weight (kg hl^-1^) |
| --- | --- | --- | --- | --- | --- | --- | --- | --- | --- | --- |
| Achille | 2.70 | 3.39 | 6.09 | 0.44 | 0.67 | 1.11 | -0.41 | 1:1.51 | 15.15 | 78.73 |
| Antalis | 4.29 | 2.34 | 6.62 | 0.70 | 0.46 | 1.16 | 0.41 | 1.51:1 | 14.98 | 79.15 |
| Aureo | 2.03 | 2.91 | 4.95 | 0.60 | 0.58 | 1.18 | 0.04 | 1.04:1 | 20.30 | 68.85 |
| Claudio | 4.14 | 2.48 | 6.62 | 0.63 | 0.49 | 1.12 | 0.25 | 1.28:1 | 15.50 | 80.50 |
| Marco Aurelio | 3.76 | 2.75 | 6.51 | 0.66 | 0.54 | 1.21 | 0.19 | 1.21:1 | 17.08 | 71.55 |
| Natur | 2.04 | 2.97 | 5.01 | 0.43 | 0.58 | 1.01 | -0.33 | 1:1.39 | 16.90 | 71.75 |
| Nazzareno | 2.70 | 2.88 | 5.58 | 0.50 | 0.57 | 1.07 | -0.15 | 1:1.16 | 16.38 | 78.40 |
| Odisseo | 2.63 | 2.82 | 5.45 | 0.46 | 0.56 | 1.02 | -0.20 | 1:1.21 | 16.00 | 74.90 |
| Rangodur | 2.51 | 3.36 | 5.88 | 0.51 | 0.67 | 1.18 | -0.26 | 1:1.29 | 16.83 | 72.25 |
| SanCarlo | 1.91 | 2.97 | 4.89 | 0.44 | 0.59 | 1.02 | -0.30 | 1:1.35 | 18.13 | 73.80 |
| Svevo | 3.51 | 2.46 | 5.96 | 0.68 | 0.48 | 1.17 | 0.34 | 1.40:1 | 17.25 | 77.18 |
| Tirex | 3.49 | 2.49 | 5.98 | 0.73 | 0.49 | 1.22 | 0.39 | 1.47:1 | 15.95 | 76.85 |

^1^ The yield of Rumbo as a pure crop (5.07 Mg ha^-1^) did not differ from that of the other two faba bean varieties in 2019.
